# Unique properties of titanium dioxide quantum dots assisted regulation of growth and biochemical parameters of *Hibiscus sabdariffa* plants

**DOI:** 10.1186/s12870-024-04794-2

**Published:** 2024-02-16

**Authors:** Reda E. Abdelhameed, Hanan Abdalla, Manar A. Ibrahim

**Affiliations:** 1 Botany and Microbiology Department, Faculty of Science, Zagazig University, Zagazig, Sharqia 44519 Egypt; 2 Physics Department, Faculty of Science, Zagazig University, Zagazig, Sharqia 44519 Egypt

**Keywords:** Titanium dioxide quantum dots, Growth, Chlorophyll, Peroxidase, Phenolics, *Hibiscus sabdariffa*

## Abstract

Owing to the uniqueness of quantum dots (QDs) as a potential nanomaterial for agricultural application, hence in the present study, titanium dioxide quantum dots (TiO_2_ QDs) were successfully synthesized via sol-gel technique and the physico-chemical properties of the prepared TiO_2_ QDs were analyzed. Based on the results, the TiO_2_ QDs showed the presence of anatase phase of TiO_2_. TEM examination revealed spherical QDs morphology with an average size of 7.69 ± 1.22 nm. The large zeta potential value (-20.9 ± 2.3 mV) indicate greater stability of the prepared TiO_2_ QDs in aqueous solutions. Moreover, in this work, the application of TiO_2_ QDs on *Hibiscus sabdariffa* plants was conducted, where *H. sabdariffa* plants were foliar sprayed twice a week in the early morning with different concentrations of TiO_2_ QDs (0, 2, 5, 10, 15 and 30 ppm) to evaluate their influence on these plants in terms of morphological indexes and biochemical parameters. The results exhibited an increasing impact of the different used concentrations of TiO_2_ QDs on morphological indexes, such as fresh weight, dry weight, shoot length, root length, and leaf number, and physio-biochemical parameters like chlorophyll a, chlorophyll b, carotenoid contents, total pigments and total phenolic contents. Remarkably, the most prominent result was recorded at 15 ppm TiO_2_ QDs where plant height, total protein and enzymatic antioxidants like catalase and peroxidase were noted to increase by 47.6, 20.5, 29.5 and 38.3%, respectively compared to control. Therefore, foliar spraying with TiO_2_ QDs positively serves as an effective strategy for inducing optimistic effects in *H. sabdariffa* plants.

## Introduction

 Quantum dots (QDs) represent a special class of semiconductor nanoparticles (NPs) with diameters in the range of 1–10 nanometers [1]. Due to their distinctive qualities, which range from optical to electrical, they attracted a great deal of attention. They are now widely employed for a variety of novel applications in biology, chemistry, medicine and agriculture and have the potential to significantly alter the world of industry [2]. It has been discovered that their chemical and physical characteristics differ from those of bulk materials [3–5]. The high surface-to-volume ratio and high reactivity of the generated QDs can improve a number of applications, including fuel cells, solar cells, photocatalysts and antibacterial activities [5].

Due to the increasing demand and use of QDs in a variety of applications, there is now a chance that these semiconducting QDs will accumulate and leak into the environment, with potentially different effects on human and environmental health and safety. Thus, there is a greater need to accurately analyze the consequences of QDs exposure on biota. Wang and Nowack [6] displayed that the majority of QDs enter the environment mostly through indirect pathways (such as dispersion after use). However, these materials can clearly interact with biota and be active within trophic food chains; these processes can modify and/or amplify their effects [7] and this kind of interaction may become a crucial when the materials are used to products of interest in agriculture and food production.

Numerous studies have demonstrated that the influences of nanomaterials on plant growth of various species are closely related to the type of nanomaterial and plant varieties [8]. QDs can be applied to plant cultivation, agricultural production and life activities as a nanofertilizer [9, 10]. It may be possible for plant cells to uptake QDs to increase and optimize solar energy trapping, potentially increasing plants’ photosynthetic efficiency [11]. The quantum size of QDs allows their easy penetration into plant cells for smart delivery. Additionally, it has been found that QDs have a major impact on plant physiological processes. QDs can stimulate photosynthesis even at low concentrations, which leads to improved plant growth and an increase in yield [12]. According to Kasibabu et al. [13] QDs are also effective in detecting and eradicating plant disease caused by bacteria and fungi without affecting normal plant growth.

Since the process of their transportation is fully established, QD uses in plants are increasing. Depending on the method of application, the plant may absorb QDs from the soil or foliar areas, where they may be exposed to the leaves or roots and internalized by the plant. When leaves are exposed, they may enter through the leaf stoma, travel through the vascular system of the leaves, and then be transported through the phloem to other sections of the plant. In contrast, for root exposure; they must be absorbed by the root, then passed via the epidermis and endodermis, enter the xylem vessel, and finally reach the plant’s aerial sections. Before they can enter the plant cell, they must pass through the cytoplasmic membrane and the cell wall. Those are expected to pass through and reach the plasma membrane whose size will be smaller than the largest pore, while the larger particles will not enter the plant cells [14].

Numerous studies have shown the beneficial effects of various QDs on plants (Table 1). For example, graphene QDs increased the growth characteristics (leaves, roots, shoots, flowers, and fruits) of treated coriander and garlic seeds [12]. Carbon dots improved seed germination, root elongation, carbohydrate production and disease resistance in addition to increasing rice yield through increased activity of the enzyme ribulose-1,5-bisphosphate carboxylase (RuBisCO) and also through better thionin gene expression [15]. Gohari et al. [16] showed that the growth of grapevine (*Vitis vinifera*) plants can be enhanced under salinity stress conditions by the application of putrescine-functionalized carbon QDs (Put-carbon QDs). On contrary, CdSe/ZnS QDs showed negative effect on the *Arabidopsis thaliana* [17]. Beside, Das et al. [18] utilized CdS:Mn/ZnS QDs to treat snow pea (*Pisum sativum*) plants, and observed a substantial decrease in total chlorophyll content, leading to phytotoxicity, at concentrations more than 40 µg mL^−1^. Consequently, to improve the safety and risk assessment of these nanomaterials, further research is required to understand the link between various NP types and plant species and their distinct effects on those species [19].

**Table 1:** A list of the studies showing the types and effects of various QDs on plants

Reference	Type of QDs	Plant	Effects
Chakravarty et al. [12]	Graphene QDs	Coriander and garlic plants	Graphene QDs increased the growth characteristics (leaves, roots, shoots, flowers, and fruits) of treated coriander and garlic seeds.
Li et al. [15]	Carbon dots	Rice plants	Carbon dots improved seed germination, root elongation, carbohydrate production and disease resistance in addition to increasing rice yield.
Gohari et al. [16]	Put-carbon QDs	Grapevine (*Vitis vinifera* cv. ‘Sultana’)	Put- carbon QDs (10 mg L^−1^) concentration, improving leaf fresh and dry weights, K^+^ content, photosynthetic pigments, chlorophyll fluorescence, proline content, total phenolics and antioxidant enzymatic activities of grapevine.
Navarro et al. [17]	CdSe/ZnS QDs	*Arabidopsis thaliana* plants	Plants exposed to CdSe/ZnS QDs suspensions experienced oxidative stress.
Das et al. [18]	CdS:Mn/ZnS QDs	Snow pea (*Pisum sativum* L.)	Snow pea seed germination and growth processes were promoted at concentration up to 40 µg mL^−1^ CdS:Mn/ZnS QDs and above this threshold, drastic reduction in seedling growth was observed as well as negative impact on total chlorophyll (a + b) content.
Liang et al. [19]	ZnO QDs	Lettuce plants	Lettuce treated with 50 to 200 mg·L^–1^ ZnO QDs, promoted Ca, Mg, Fe, Mn, Zn, and B absorption and accumulation; increased soluble sugar content; and improved the lettuce biomass and nutritional quality. While, lettuce treated with 500 mg·L^–1^ ZnO QDs produced a large amount of ROS, which adversely affected the absorption of nutrients, soluble protein content, and chlorophyll content, thus reducing plant biomass.
Gong and Dong [20]	Cerium-doped carbon QDs	Wheat plants	Cerium-doped carbon QDs (0.025 mg mL^–1^) promoted the growth and development of wheat plants.
Li et al. [21]	SiQDs	Italian lettuce	SiQDs significantly promoted Italian lettuce seedling growth (root length, seedling height and biomass) at concentrations below 30 mg L^−1^ by increasing the chlorophyll *a* and *b* content, soluble sugar and water content.
Feng et al. [22]	Graphene QDs	Mung bean and tomato seedlings	Graphene QDs enhanced the accumulation of chlorophyll in mung bean (250–1250 mg L^−1^) and tomato (250–500 mg L^−1^) seedlings after exposure for 2 weeks. High concentrations of graphene QDs (1000–1500 mg L^−1^) led to an increase in the H_2_O_2_, malondialdehyde, proline, glutathion levels, as well as increased catalase and glutathione reductase activities in seedlings of both species.
Haydar et al. [23]	Fe–Mn graphene QDs	*Triticum aestivum*	Fe–Mn graphene QDs application increased enzymatic antioxidants like catalase, peroxidase, glutathione reductase and total phenolics of *T. aestivum* plants under normal and stress condition.

Titanium dioxide (TiO_2_) is an important semiconductor oxide because of its strong photocatalytic capabilities, low cost, eco-friendliness and non-toxicity [24]. Its photocatalytic activity can also be further increased using a variety of physical and chemical techniques. Because of these qualities, TiO_2_ is a promising material for a wide range of applications and it being one of the most widely used NPs in agriculture [25, 26]. It has been discovered that TiO_2_ NPs increase enzyme activity and stimulate plant growth in some plants [27–30]. While, TiO_2_ in the form of QDs exhibit markedly increased surface area due to small particle size [4].

In Egypt, the roselle plant, *Hibiscus sabdariffa*, is referred to as “Karkadeh” [31] and is a member of the Malvaceae family. This plant has the potential to be grown in the summer as an industrial and medicinal plant and acts as an affluent source of phenolic compounds. Furthermore, *H. sabdariffa* L. and its extract have been shown to have a number of medical benefits worldwide, including the treatment of kidney stones, hypertension, improving the digestive system, preventing cancer and protecting liver damage [32, 33]. Much research has been done on the role of various NPs in diverse crop plants to understand their physiological and biochemical impacts, including both antagonistic and synergistic effects [29, 34]. Nevertheless, there are currently few findings about the function of QDs in plants, particularly *Hibiscus* plants. To our knowledge, up to date, there are no research studies on the effect of TiO_2_ QDs on the physiological and biochemical properties of plants. In this work, sol-gel method was used to prepare TiO_2_ QDs and the prepared QDs were characterized by various techniques including X-ray diffraction (XRD), fourier transform infrared (FTIR), high resolution-transmission electron microscopy (HR-TEM) and zeta potential. Moreover, their application on *H. sabdariffa* plants was conducted in terms of morphological indexes and biochemical parameters.

## Materials and methods

### Preparation of TiO_2_ QDs

#### Chemicals

Titanium (IV) isopropoxide (97%, Aldrich), ethanol absolute (Adwic), nitric acid (Adwic) and deionized water. All of these chemicals were of analytical grade and used without further purification.

TiO_2_ QDs was prepared by sol-gel technique using titanium (IV) isopropoxide (TTIP) [Ti [OCH (CH_3_)_2_]_4_ as precursor in accordance with the previous report [35]. The sol-gel method includes hydrolysis and condensation process of titanium (IV) isopropoxide in aqueous media under acidic condition. First, TTIP was added to absolute ethanol at ratio of (1: 3) under continuous stirring for 30 min until a homogenous clear yellow solution formed. Then, solution of deionized water and absolute ethanol with a volume ratio of (1:4) was prepared. The pH value of the former solution was adjusted at ~ 2 by drop wise addition of nitric acid (HNO_3_) under continuous stirring for 1 h at room temperature to restrain the hydrolysis process of the solution. Finally, the prepared solution was added slowly into TTIP solution and aged under stirring for 2 h. The gel was digested at 80 °C in water bath for 1 h until most ethanol evaporate, after which dried at 80 °C for 24 h. The dry powder was then calcined at 400 °C for 2 h to obtain TiO_2_ QDs.

### TiO_2_ QDs characterization

X-ray diffraction (XRD) patterns were documented in the 2θ range of 4–70° with a Pan Analytical Model X’ Pert Pro which was equipped with CuKα radiation (λ = 0.1542 nm). An accelerating voltage of 40 kV and an emission current of 40 mA were used. Using an FTIR spectrometer model (JASCO, FT/IR–4100 type A), the fourier transform infrared (FTIR) spectrum was acquired at room temperature in the wavenumber range of 400–4000 cm^−1^. Dual split beam UV–Vis spectrophotometer (Model Spectro dual split beam, UVS-2700) was used to measure UV–Vis spectrum in the wavelength range from 270 to 700 nm with 1 nm step.

High Resolution Transmission Electron Microscopy (HR-TEM) image for the prepared sample was recorded on a JEOL JEM-1230 electron microscope operating at an acceleration voltage of 120 kV to verify the QD size and morphology. Using dynamic light scattering methods (Zeta sizer, Malvern, UK), the zeta potential of TiO_2_ QDs was determined. Double-distilled water was used to dilute the sample 1:100, and the measurement was conducted at 25 °C with a 90 ° detection angle.

### Preparation of stock solutions of TiO_2_ QDs

Stock solution was prepared by dissolving 1000 mg TiO_2_ QDs in litre distilled water at room temperature. The solution was subjected to a 2 h sonication process in a Branson Model B200 ultrasonic bath sonicator to guarantee dispersion and prevent clumping and agglomeration. Then different concentrations were prepared from the stock solution as shown in Table 2.

**Table 2:** Treatments and concentration of TiO_2_ QDs

Treatments	Concentration
T0	0 ppm TiO_2_ QDs
T1	2 ppm TiO_2_ QDs
T2	5 ppm TiO_2_ QDs
T3	10 ppm TiO_2_ QD
T4	15 ppm TiO_2_ QD
T5	30 ppm TiO_2_ QD

### Field experiment and culture of the plants

*H. sabdariffa* L. seeds were bought after permission from the Agricultural Research Center, Giza, Egypt. Experiment was carried out a field at a Faculty of Science, Zagazig University, Egypt. *H. sabdariffa* seeds were disinfected with 5% sodium hypochlorite solution for 10 min and then were sowed in the field. After 15 days from sowing, TiO_2_ QDs were sprayed twice a week using a conventional hand sprayer in the early morning. After 15 days from TiO_2_ QDs application, samples were collected at the vegetative stage.

### Plant morphological indexes

Samples from each treatment were taken at the vegetative stage, following 30 days of cultivation, and properly cleaned with tap water to get rid of dust and dirt particles. To measure morphological indexes of *Hibiscus* plants treated with different TiO_2_ QDs concentrations, plants were separated to roots and shoots and root length, shoot length and plant height were measured using a measuring scale and expressed in cm. Also the fresh and dry weights of roots and shoot were measured using electronic balance and expressed in gram. Dry weights were measured after oven dried at 60 °C for 2 days. Number of leaves was counted for each treatment. At harvest, fruits were collected.

### Plant biochemical analysis

#### Determination of photosynthetic pigments

In the early morning, samples of fresh *Hibiscus* leaves were randomly taken from each treatment to evaluate the pigments found in leaves, specifically chlorophyll a, chlorophyll b and carotenoids so 100 mg of fresh leaves were extracted using 10 mL of 85% acetone. Using pure 85% acetone as a blank, the produced color was measured at wavelengths of 663, 644, and 452.5 nm using a UV-visible spectrophotometer, RIGOL (Model Ultra-3660), as described by Metzner et al. [36]. Calculations of carotenoid, chlorophylls a and b contents were performed with consideration for the dilution factor. The following formulae were used to calculate the pigment fraction concentration in mg mL^−1^.

1 $$\mathrm{Chlorophyll}\;\mathrm a\;=10.3\mathrm E663-0.918\mathrm E644$$

2 $$\mathrm{Chlorophyll}\;\mathrm b\;=19.7\mathrm E644-3.87\mathrm E663$$

3 $$\mathrm{Carotenoids}\;=4.2\mathrm E452.5-(0.0264\;\mathrm{Chl}\;\mathrm a+0.426\;\mathrm{Chl}\;\mathrm b)$$

Finally, the pigment fractions were calculated as µg/mg fresh wt. of leaves.

### Assessment of total soluble protein content

Using mortar and pestle under ice-cold conditions, 10 mL of the extraction buffer containing 100 mM potassium phosphate (pH 7.0), 0.1 mM EDTA and 1% (w/v) polyvinyl pyrrolidone were added to a known fresh wt of *Hibiscus* leaf (1 g) in order to homogenize the protein according to the Qiu et al. [37] method. After centrifugation (10,000 rpm for 20 min) at 4 °C, the supernatant was collected to evaluate total soluble proteins and antioxidant enzymes (peroxidase; POX and catalase; CAT). The protein content from each treatment was calculated following the method of Lowry et al. [38] with a few changes. The mixture was added to the alkaline copper sulphate reagent and shaken for 10 min. After adding Folin’s reagent, the mixture was kept in an incubator for 30 min. Every sample’s absorbance was measured at 700 nm in comparison to a blank. Using the reference curve of bovine serum albumin as a standard, the concentration of total soluble proteins was calculated and represented as mg/g fresh wt.

### Assessment of total phenolic compounds

First, 0.5 g of *Hibiscus* plants was extracted with 10 mL of 80% methanol for 30 min. and the extract was then centrifuged at 8000 rpm for 30 min. The supernatant was collected and the **t**otal phenolics were determined spectrophotometrically using Folin–Ciocalteau reagent [39]. Briefly 1.4 mL of distilled water and 0.1 mL of 50% Folin-Ciocalteu phenol reagent (diluted with water 1:1), 0.5 mL of the extract was added. After 3 min of incubation in darkness at room temperature, 125 µL of 20% Na_2_CO_3_ was added and mixed. The spectrophotometer was used to measure the blue complex’s absorbance at 725 nm. Through the use of a standard curve created using various gallic acid concentrations, the absorbance readings were converted to total phenols and expressed as µg gallic acid equivalent\g fresh wt.

### Assay of antioxidant enzyme

Aebi [40] and Chance and Maehly [41] protocols outline measurement of CAT and POX activity in plant tissue by spectrophotomeric assay. CAT activity in the supernatant was determined by monitoring H_2_O_2_ consumption for 2 min at 240 nm. In order to determine POX, 4 mL of the assay mixture; which included 1 mL of enzyme extract, 50 µM catechol, 50 µM H_2_O_2_, and 300 µM phosphate buffer (pH 6.8) were prepared and optical density was then measured at 470 nm.

### Statistical analyses

Excel and SPSS software were used for the statistical analysis of the experimental data (one way analysis of variance, ANOVA). The mean ± standard error of three replicates was utilized to express all estimated parameters. Different letters indicate significant differences according to Duncan’s multiple range test which calculated at the 0.05 level of significance (*p* < 0.05).

## Results and discussion

### Characterization of TiO_2_ QDs

**Fig. 1:**
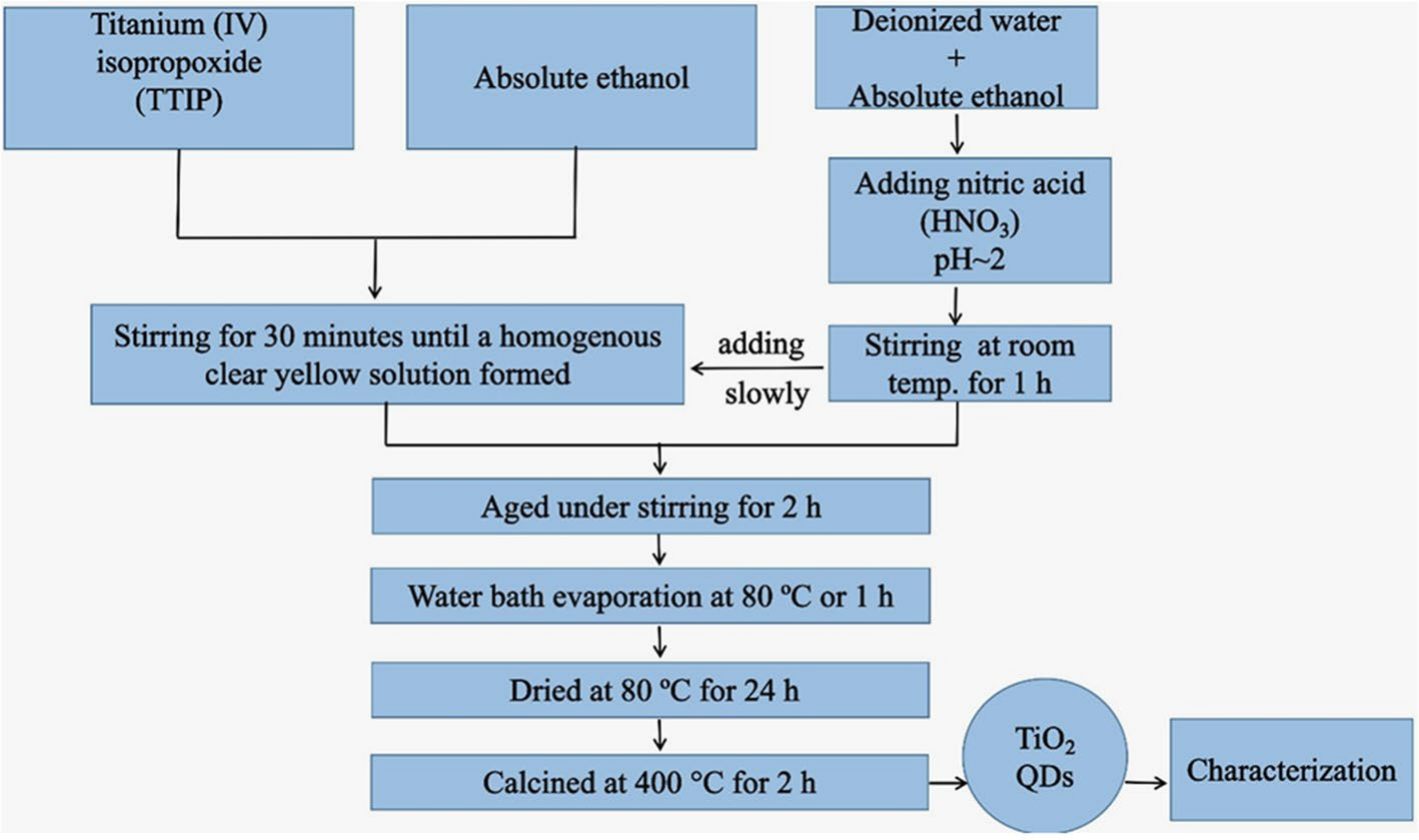
Flow chart for the synthesis of TiO_2_ QDs from titanium (IV) isopropoxide via sol-gel process

**Fig. 2:**
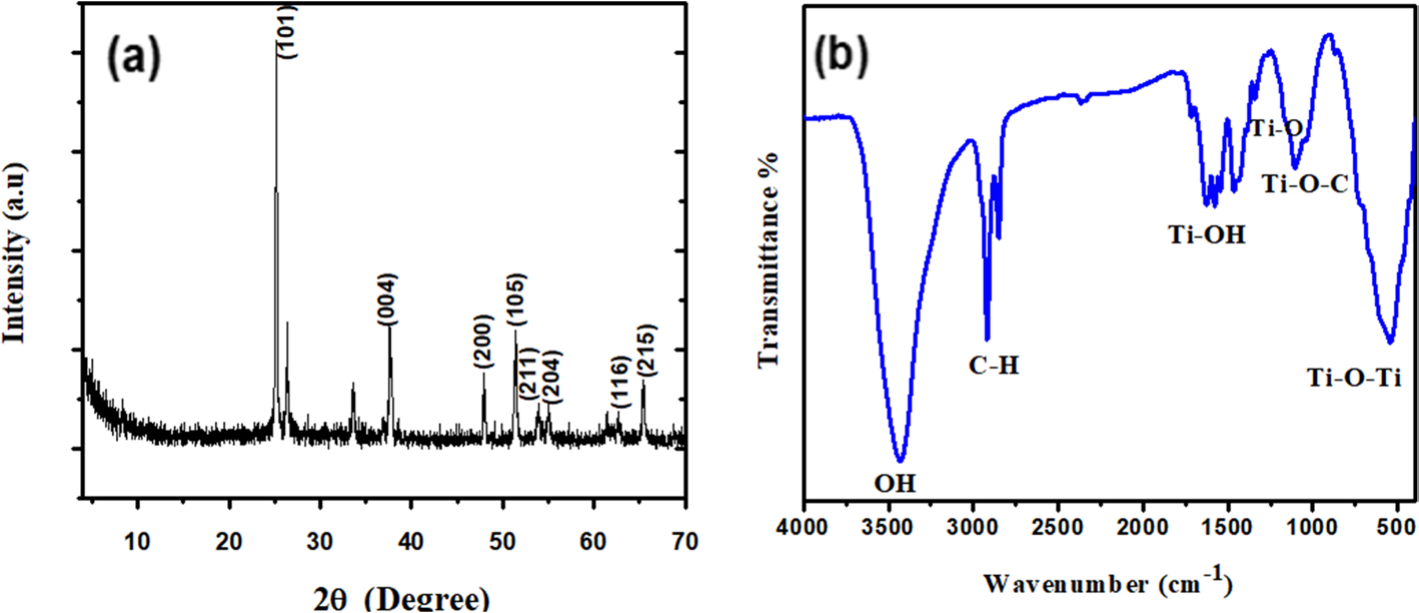
**a** X-ray diffraction pattern and **b** FTIR spectrum of TiO_2_ QDs

Figure 1 showed the flow chart for the synthesis of TiO_2_ QDs from titanium (IV) isopropoxide via sol-gel process. To further understand the crystalline properties of the prepared TiO_2_ QDs, X-ray diffraction (XRD) was utilized. The patterns of XRD were investigated in the range of 2θ between 4° and 70°. The XRD pattern of the TiO_2_ QDs calcinated at 400 °C is presented in Fig. 2a. All the diffraction peaks of the XRD pattern were clearly indexed to anatase phase (JCPDS No. 21–1272) with tetragonal structure. The diffraction peaks located at 2θ = 25.21°, 37.60°, 47.94°, 51.45°, 53.92°, 55.01°, 62.59° and 65.42° corresponding to the diffraction planes (101), (004), (200), (105), (211), (204), (116) and (215), respectively. Using Debye-Scherrer equation [42] for the most intense (101) diffraction peak;

4 $$\mathrm D=\mathrm{K}\lambda/\,\mathrm\beta\,\cos\,\mathrm\theta$$

where k is constant related to the crystallite shape (K ≈ 1), λ represents the X-ray wavelength (1.5406 Å), β is FWHM (Full Width at Half Maximum) of the high intensity peak, and θ Bragg’s angle of diffraction. The crystallite size was computed to be ~ 7.2 nm thereby confirming formation of the TiO_2_ QDs.

The generated TiO_2_ QDs’ functional groups have been verified by the FTIR spectra (Fig. 2b). There is a band present at 3430 cm^−1^ in the sample due to OH stretching vibrations. Two bands at 2918.7 and 2851 cm^−1^ could be ascribed to the characteristic frequencies of residual organic species, which was not completely removed by distilled water washing are assigned to C-H stretching vibrations. The band present in the sample at 1625 cm^−1^ shows Ti-OH bending vibrations of adsorbed H_2_O molecules and band at 1341 cm^−1^ indicating Ti-O [43]. The weak band observed at 1106 cm^−1^ belongs to the Ti–O–C group. The band present at 542.8 cm^−1^ represents Ti–O–Ti stretching bonding [3].

**Fig. 3:**
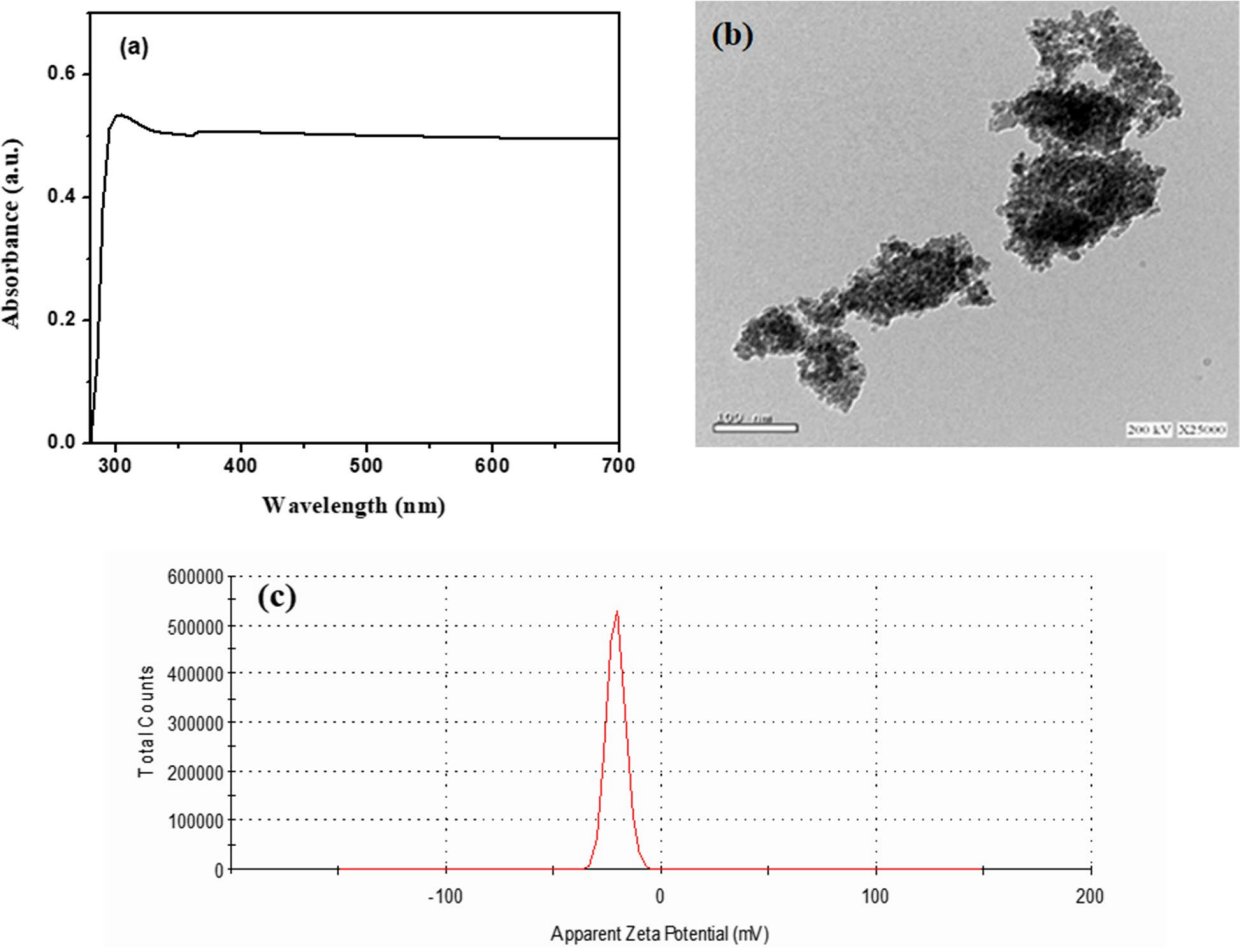
**a** UV-Vis absorption spectrum, **b** TEM image and **c** Zeta potential of TiO_2_ QDs

The UV–Vis spectrum for TiO_2_ QDs is presented in Fig. 3a. The absorption curve of the TiO_2_ QDs has a peak in the UV region at around 300 nm, which is lower than that for anatase TiO_2_ NPs. The reduction in the TiO_2_ size to ~ 7 nm induced a blue shift in the onset of absorption, this in agreement with previous study reported by Javed et al. [44].

Figure 3b shows the TEM image of the prepared TiO_2_ QDs which clearly shows the random distributions of homogenous fine spherical shaped particles with average diameter of 7.69 ± 1.22 nm, which is fairly consistent with calculated average crystalline size from XRD data. A slight agglomeration of particles can be observed, possibly due to the hydrophilic and high-energy surface of the NPs during the drying process. Such small nanosize makes it possible for them to enter the plant body [45].

Zeta potential is an essential measure which reflects the degree of repulsive force among particles and the stability of dispersion. Zeta potential is necessary for controlling the stability of TiO_2_ QDs in suspensions. Figure 3c shows that the TiO_2_ QDs had a large zeta value (-20.9 ± 2.3 mV), illustrating the negative surface charge of TiO_2_ QDs and indicating their greater stability in aqueous solutions. This also suggests that the TiO_2_ QDs repelled each other and have a low possibility to agglomerate [46]. Thus at neutral pH, TiO_2_ QDs dispersed in water have negatively charged surface that can adsorb cationic dyes and other organic pollutants on their surface through electrostatic attraction [47].

### Effect of TiO_2_ QDs on growth of *Hibiscus* plants

**Fig. 4:**
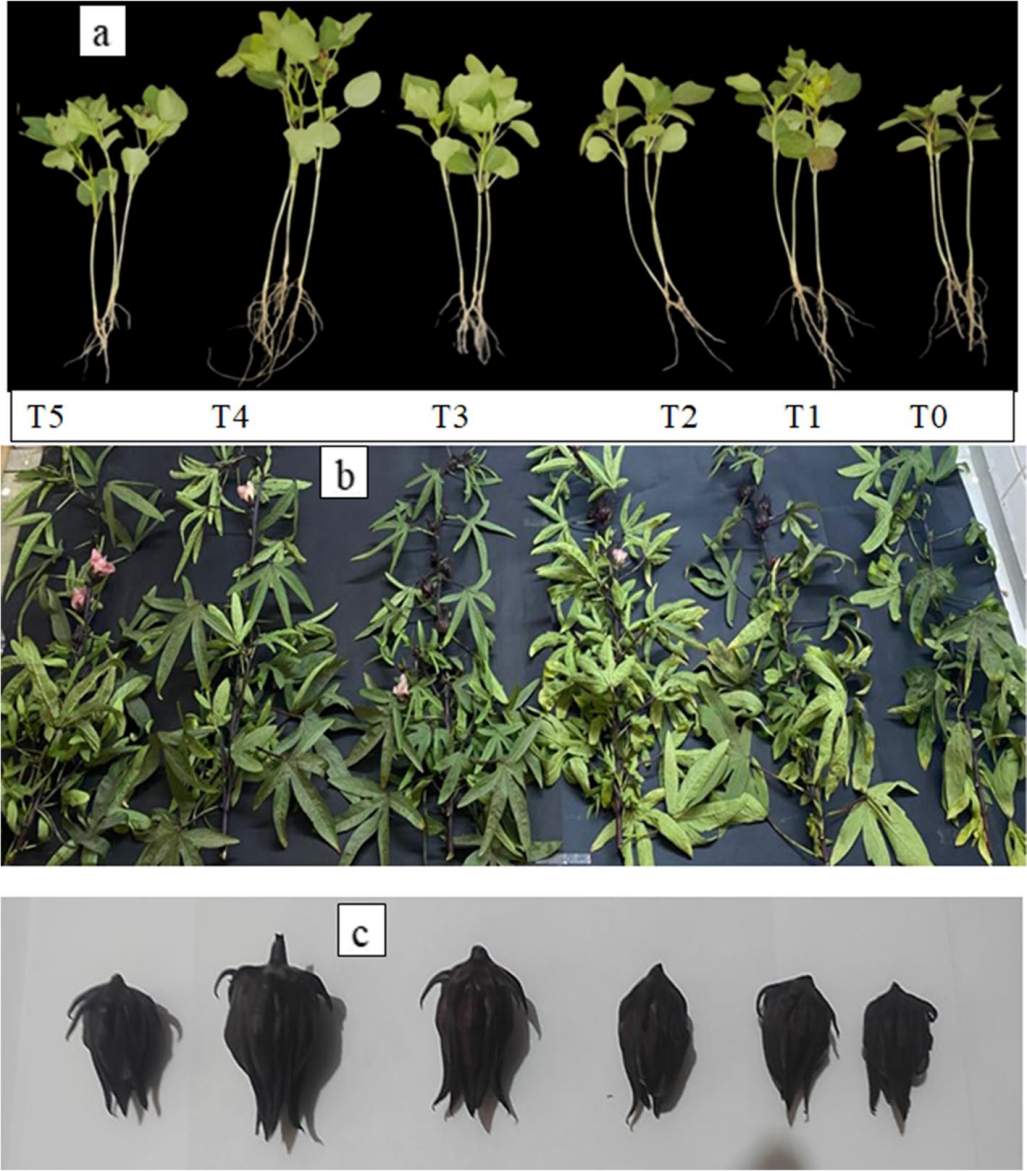
*Hibiscus* plants under the effect of different concentrations of TiO_2_ QDs; **a** at vegetative stage, **b** at flowering stage and **c** the obtaining fruits

**Fig. 5:**
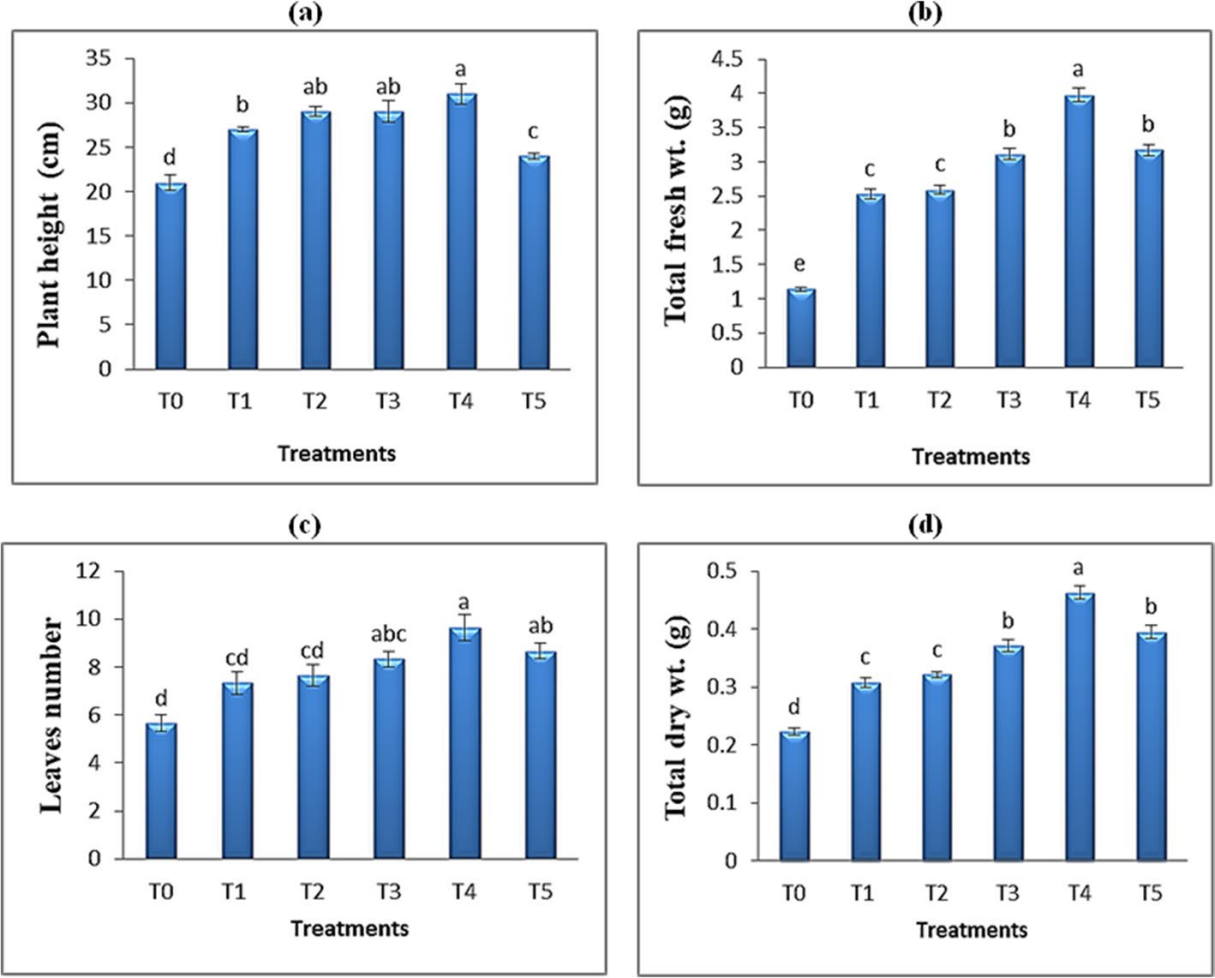
Effect of different concentrations of TiO_2_ QDs foliar spray on growth of *Hibiscus* plants in terms of **a** plant height (cm), **b** total fresh wt. (g), **c** leaves number and **d** total dry wt. (g). Data are the mean of three replicates ± standard error (error bars, *n* = 3). Different letters above bars indicate a significant difference between treatments using ANOVA followed by Duncan’s multiple range test (*p* < 0.05)

Various nano-sized materials involving QDs have been employed to promote plant growth because of their improved biocompatibility and low toxicity [48]. To study the impact of foliar spraying with different concentrations of TiO_2_ QDs, the weights (fresh and dry) and lengths of roots and shoots and leaf numbers of *Hibiscus* plants were observed and their data was calculated. Generally, all growth characters of *Hibiscus* plants such as shoot length, root length, fresh wt. and dry wt. of shoot and roots were increased as a result of foliar spray with TiO_2_ QDs (Fig. 4; Table 3). The highest values of *Hibiscus* growth parameters were obtained from the concentration of 15 ppm TiO_2_ QDs. Compared with the control group, the plant height of *Hibiscus* plants treated with 15 ppm TiO_2_ QDs was increased by 47.6% (Fig. 5). Similarly, Gong and Dong [20] demonstrated that, at a specific concentration range, carbon QDs or cerium-doped carbon QDs stimulate wheat growth and development. Chakravarty et al. [12] have also reported using graphene QDs to boost the growth of garlic and coriander plants; where during planting, the QDs were applied, and the plants’ roots, leaves, and flowers appeared to grow longer and heavier. These results are in an agreement with those obtained by Abdalla et al. [29] who found that TiO_2_ NPs had significant and consistent effect on the germination and growth of soybean plants under normal and salinity stress condition. More crucially, Li et al. [21] found that, at concentrations below 30 mg L^−1^, the QDs greatly boosted the growth of Italian lettuce seedlings in terms of biomass, root length, and seedling height. This effect may have been caused by an increase in soluble sugar and water content.

**Table 3:** Effect of different concentrations of TiO_2_ QDs on growth of *Hibiscus* plants at the vegetative stage

Treatments	Shoot length (cm)	Root length (cm)	Shoot fresh wt. (g)	Root fresh wt. (g)	Shoot dry wt. (g)	Root dry wt. (g)
**T0**	13.66 ± 0.6c	7.33 ± 0.33c	1.04 ± 0.027e	0.096 ± 0.0025d	0.202 ± 0.005d	0.0206 ± 0.0005d
**T1**	16.66 ± 0.33b	11 ± 0.57ab	2.39 ± 0.063c	0.14 ± 0.0037c	0.281 ± 0.007c	0.0275 ± 0.00073b
**T2**	17 ± 1.15b	12 ± 0.57a	2.44 ± 0.038d	0.141 ± 0.0034c	0.301 ± 0.005bc	0.0269 ± 0.00064bc
**T3**	17.5 ± 1.15ab	11.5 ± 0.33a	2.97 ± 0.078b	0.138 ± 0.006c	0.347 ± 0.009b	0.0251 ± 0.00066c
**T4**	19.67 ± 0.88a	11.33 ± 0.57a	3.77 ± 0.099a	0.211 ± 0.0056a	0.426 ± 0.011a	0.0367 ± 0.00097a
**T5**	16.3 ± 0.33b	8 ± 0.29b	3 ± 0.099b	0.175 ± 0.008b	0.367 ± 0.0097b	0.0277 ± 0.00073b

Data are the mean of three replicates ± standard error (*n* = 3). For each parameter, different letters in the same column indicate a significant difference between treatments using ANOVA followed by Duncan’s multiple range test (*p* < 0.05)

Various nanomaterials have huge applications because of their distinct characteristics, which include enhanced cellular penetration, fast distribution inside organisms, and strong biochemical reactivity. NPs can quickly enhance the plant’s characteristics because of their capacity to diffuse into the plant cell. Particles that are smaller in diameter than the cell wall’s pore diameter are typically able to pass through the plasma membrane [49, 50]. Recently, Arshad et al. [48] demonstrated that the special qualities of graphene QDs allow for their application in nanobiotechnology, where they can be employed as a regulator of plant growth because of their smaller size than that of the cell wall.

### Effect of TiO_2_ QDs on chlorophyll contents of *Hibiscus* plants

**Fig. 6:**
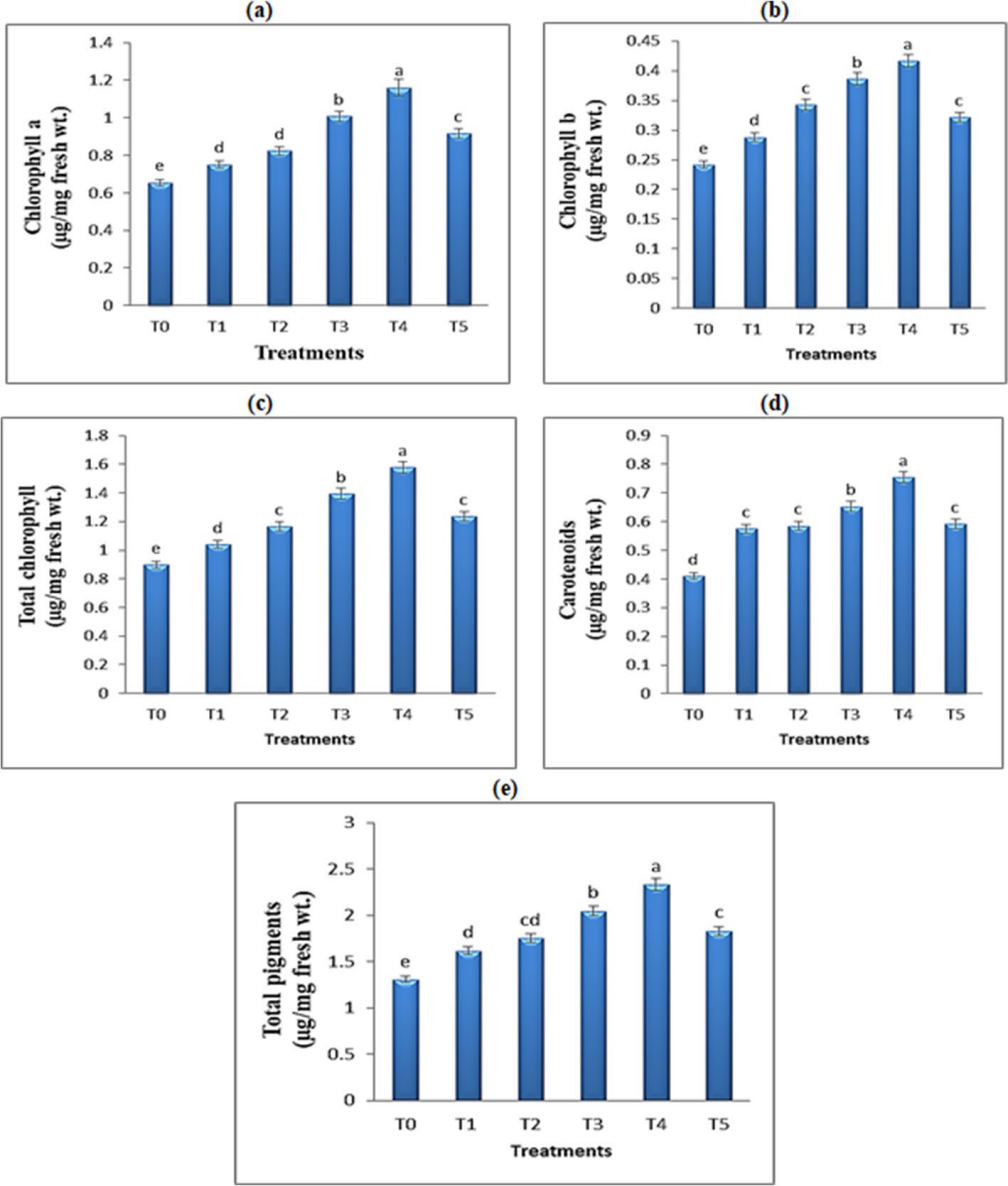
Effect of different concentrations of TiO_2_ QDs on pigment fractions of *Hibiscus* plants. Data are the mean of three replicates ± standard error (error bars, *n* = 3). Different letters above bars indicate a significant difference between treatments using ANOVA followed by Duncan’s multiple range test (*p* < 0.05)

Plants employ a class of green pigments called chlorophyll to absorb light energy. An excess of chlorophyll can boost a plant’s ability to photosynthesize, activate its photosystem, and produce carbohydrates [51] and thus promote their growth and development. It has been noted that specific nanomaterials can stimulate the physiological processes in plants such as photosynthesis which attracted more and more attention [21, 52, 53]. The application of varying concentrations of TiO_2_ QDs solution to *Hibiscus* plants resulted in a notable increase in pigment content in their leaves, as illustrated in Fig. 6a-e and this suggests that the spraying process may have a positive impact on the production of chlorophyll. Giving highly significant result was at 15 ppm which recorded an increase of 77.2, 72.1 and 83.6% for chlorophyll a, b and carotenoids contents, respectively as compared with the untreated plants. Similarly, following treatment with 0.025 mg mL^−1^ of cerium-doped carbon QDs, the amount of chlorophyll in the leaves of wheat seedlings increased by 51% as compared to the control group [34]. Interestingly, by using different concentrations of SiQDs, Li et al. [21] observed that the chlorophyll a and b contents increased with no inhibition, even at the highest dose of 200 mg L^−1^. The study of Gohari et al. [16] documented benefits of carbon QDs in chlorophyll a and b contents under normal and salinity conditions. Further investigation revealed that carbon QDs can enhance the photosystem activity by improving the electron transfer rate and also affect other critical indicators in photosynthesis, such as chlorophyll content and motivates RuBisCO activity [54]. QDs convert a given fraction of the solar spectrum into one that can be more effectively utilized by photosynthetic light reactions so; QDs provide an efficient and cost-effective light source alternative with proper spectral composition to intensify the photosynthetic rate of plants [55, 56].

According to a study by Moaveni et al. [57], TiO_2_ NPs can increase pigment content and facilitate the transportation of photosynthetic materials by recovering chlorophyll structure and light sorption. Furthermore, the research conducted by Liang et al. [19] shown a considerable increase in chlorophyll at 50 mg L^−1^ ZnO QDs. However, when exposed to 500 mg L^−1^ ZnO QDs, there was a significant decrease in the amount of chlorophyll, which may have been caused by an increase in malondialdehyde content that damaged the chloroplast membrane and reduced the amount of chlorophyll.

### Effect of TiO_2_ QDs on the total soluble protein content of *Hibiscus* plants

**Fig. 7:**
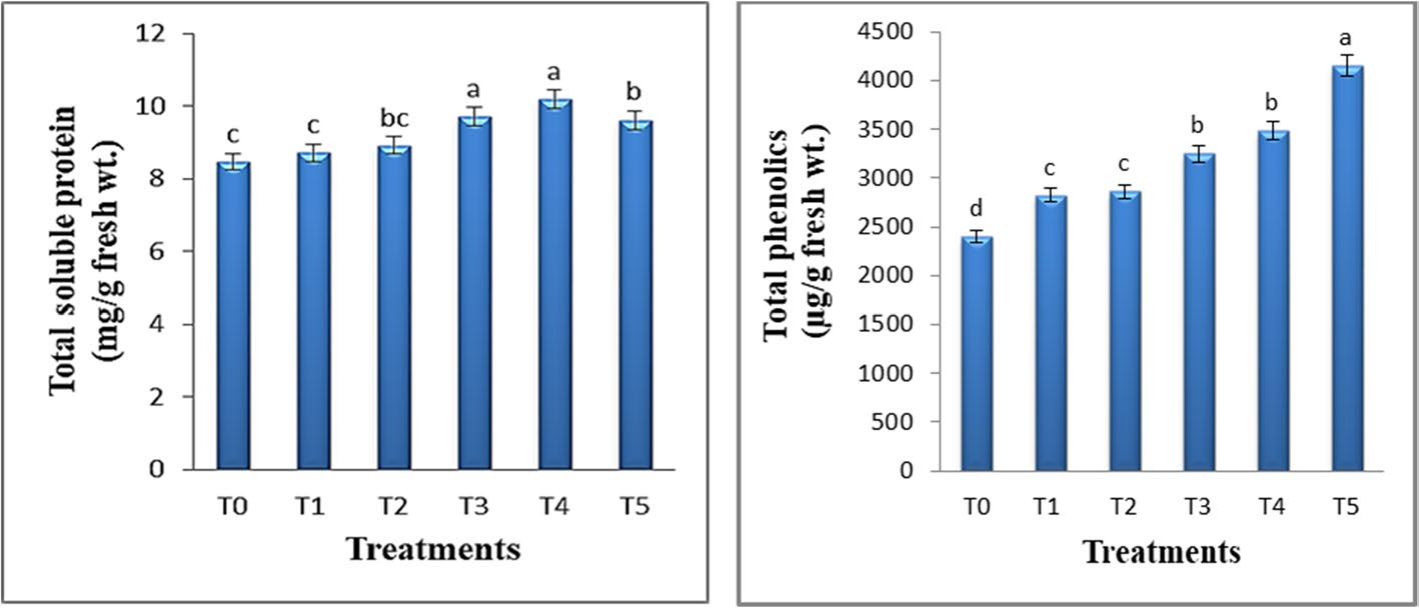
Effect of different concentrations of TiO_2_ QDs on total soluble protein and total phenolics of *Hibiscus* plants. Data are the mean of three replicates ± standard error (error bars, *n* = 3). Different letters above bars indicate a significant difference between treatments using ANOVA followed by Duncan’s multiple range test (*p* < 0.05)

Because of their small size, higher surface area and absorption rate and sufficient reactive sites, NPs are employed as an emerging technique to activate specific biochemical events relevant to plant physiological output [58]. The foliar application of *Hibiscus* plants with TiO_2_ QDs at 2, 5, 10, 15 and 30 mg L^−1^ had a significantly increased the total protein content as compared with control ones (Fig. 7a). The highest values for total protein were determined for the plants treated with 10 and 15 ppm TiO_2_ QDs with an increase of 15.1 and 20.5%, respectively as compared with control. According to Liang et al.’s [19] research from 2021, at 50 and 100 mg L^−1^ ZnO QDs, respectively, the soluble protein content of lettuce plants increased by 6.12 and 50.86%. Similar results were also seen by Hu et al. [59], who discovered that treating plants with TiO_2_ NPs raised its soluble protein content. The results obtained are in line with the earlier research conducted by Sturikova et al. [60], which showed that plants treated with low-concentration ZnO QDs had a significant increase in their soluble protein and soluble sugar content. This improvement in plant nutrition may have implications for the agricultural sector. Simultaneously, soluble protein accumulation and plant growth promote antioxidation and enhance plant resilience [54]. When the plant is exposed to external stress, the amount of soluble protein content maintains a metabolic balance [61]. Also, Liang et al. [19] discovered that administering ZnO QDs at a high concentration (500 mg L^−1^) can considerably lower the amount of soluble proteins. This could be attributed to lipid degradation in the cell membrane, which hinders the production of new proteins.

### Effect of TiO_2_ QDs on the total phenolics content of *Hibiscus* plants

The concentration of phenols in plant tissues is a good indicator that enables researchers to estimate the range of tolerance to the stress factors that occur in plants [62]. Phenolic compounds are signaling molecules in the defense mechanism of plants. Plants that produce more phenolic compounds are better able to withstand oxidative stress because these compounds function as water-soluble, non-enzymatic antioxidants that restrict free radicals and reactive oxygen species (ROS) [63]. Current results (Fig. 7b) revealed that the content of total phenolics increased as a result of the applied different concentrations of TiO_2_ QDs. The highest increase in the value of this parameter (by 45.2 and 72.9%) was observed as a result of the application of the highest concentration of TiO_2_ QDs (15 and 30 ppm), compared to the control. Similarly, increased phenolics were reported by Feng et al. [22] after applying low concentrations of QDs. The findings of Gohari et al. [16] showed that carbon QDs at 10 mg L^−1^ and Put-carbon QDs at 5 and 10 mg L^−1^ were beneficial in protecting against oxidative stress caused by NaCl by increasing the production of phenolics. A study of Haydar et al. [23] observed that Fe–Mn nanocomposites doped graphene QDs increased total phenolics of *Triticum aestivum* plants under normal and stress condition. In addition, TiO_2_ NPs treatment enhanced plant phenolics under normal and salinity conditions [64]. Phenolics decrease oxidative stress and lessen its negative effects by preventing ROS production and accumulation [65, 66].

### Effect of TiO_2_ QDs on POX and CAT activities of *Hibiscus* plants

**Fig. 8:**
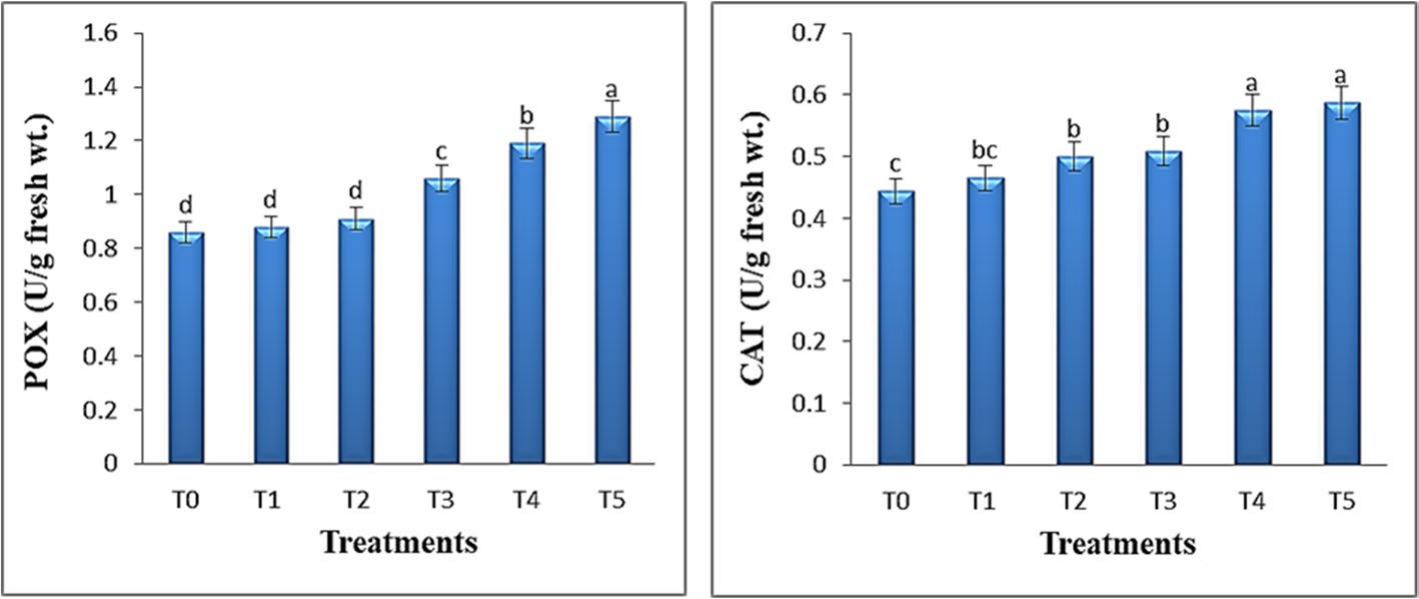
Effect of different concentrations of TiO_2_ QDs on the activity of antioxidant (POX and CAT) of *Hibiscus* plants. Data are the mean of three replicates ± standard error (error bars, *n* = 3). Different letters above bars indicate a significant difference between treatments using ANOVA followed by Duncan’s multiple range test (*p* < 0.05)

To investigate the antioxidant activity of TiO_2_ QDs in *Hibiscus* plants, the antioxidant enzymes were quantified. Plants contain large amounts of POX, an adaptable enzyme with high activity in the antioxidized system, whose activity can precisely represent plant development characteristics, metabolism, and environmental adaptation. Additionally, POX can convert H_2_O_2_ into water and safe oxygen [67, 68]. The data in Fig. 8a and b showed the effect of different concentrations of TiO_2_ QDs (0, 2, 5, 10, 15 and 30 ppm) on POX and CAT activities of *Hibiscus* plants through foliar application. As shown in this figure, there are significant increases in their activities with increasing the TiO_2_ QDs concentrations. POX and CAT activity of *Hibiscus* plants was increased by 38.3 and 29.5% at 15 ppm TiO_2_ QDs and by 50 and 32.2% at 30 ppm TiO_2_ QDs compared with the control group.

Increases in POX activity in wheat plants following the application of varying concentrations of cerium-doped carbon QDs as a nanofertilizer, as reported by Gong and Dong [20], suggest that the plant has become more acclimated to its external environment and is more resilient to stress. According to a different study by Liang et al. [19], ZnO QDs at concentrations of 50, 100, 200 and 500 mg L^−1^ have a remarkable effect on the CAT and POX activity of lettuce shoots. All concentrations increase CAT activity, but the POX activity would be ineffective at concentrations higher than 500 mg L^−1^. Similarly, Gohari et al. [16] found that under both control and stress settings, the maximum CAT activity was observed after applying 10 mg L^−1^ Put-Carbon QDs. POX with large number of isoenzymatic forms contribute in a variety of cellular functions such as growth, development, differentiation, senescence, auxin catabolism and lignifications [30, 69]. POX is able to catalyze the reaction of H_2_O_2_ with amines and acids to lessen damage to the cell membrane and maintain the membrane’s selective permeability. CAT has important functions in the growth, biotic, and abiotic stressors of plants by breaking down H_2_O_2_ into water and oxygen to prevent cellular oxidative damage [70].

## Conclusions

To sum up, the sol–gel approach was successfully used to synthesize TiO_2_ QDs with a tetragonal structure and an anatase phase. TEM confirmed that the average size of the TiO_2_ QDs was around 7 nm. From the present study, it can be inferred that foliar application of TiO_2_ QDs could influence the growth parameters of *Hibiscus* plants, thereby caused the changes in chlorophyll content, soluble protein and total phenolics. Moreover, the application of TiO_2_ QDs enhanced the antioxidant activity of *Hibiscus* plants by increasing POX and CAT activities. The results of this work show strong evidence for the high efficiency of this new nanofertilizer on *Hibiscus* growth enhancement. However, more research is needed to determine the exact mechanism by which TiO_2_ QDs affect plants under both normal and stressful environments for use in biotechnology and agriculture.

## Data Availability

No datasets were generated or analysed during the current study.

## Abbreviations

CAT: Catalase
FTIR: Fourier transform infrared
HR-TEM: High Resolution-Transmission Electron Microscopy
NPs: Nanoparticles
POX: Peroxidase
Put: Putrescine
QDs: Quantum dots
ROS: Reactive oxygen species
RuBisCO: Ribulose-1,5-bisphosphate carboxylase
TiO_2_: Titanium dioxide
TTIP: Titanium isopropoxide
XRD: X-Ray Diffraction
